# WDR5 high expression and its effect on tumorigenesis in leukemia

**DOI:** 10.18632/oncotarget.9312

**Published:** 2016-05-12

**Authors:** Zheng Ge, Evelyn J. Song, Yuka Imamura Kawasawa, Jianyong Li, Sinisa Dovat, Chunhua Song

**Affiliations:** ^1^ Department of Hematology (Key Department of Jiangsu Medicine), Zhongda Hospital, Southeast University Medical School, Nanjing, Jiangsu, China; ^2^ Department of Pediatrics, Pennsylvania State University College of Medicine, Hershey, PA, USA; ^3^ Department of Hematology, The First Affiliated Hospital of Nanjing Medical University, Jiangsu Provincial Hospital, Nanjing, China; ^4^ Pennsylvania State Hershey Genome Sciences Facility, Pennsylvania State College of Medicine, Hershey, PA, USA

**Keywords:** WDR5, H3K4me3, MLL, leukemia

## Abstract

WD repeat domain 5 (WDR5) plays an important role in various biological functions through the epigenetic regulation of gene transcription. However, the oncogenic effect of WDR5 in leukemia remains largely unknown. Here, we found *WDR5* expression is increased in leukemia patients. High expression of *WDR5* is associated with high risk leukemia; Patients with *WDR5* and *MLL*1 high expression have poor complete remission rate. We further identified the global genomic binding of *WDR5* in leukemic cells and found the genomic co-localization of WDR5 binding with H3K4me3 enrichment. Moreover, WDR5 knockdown by shRNA suppresses cell proliferation, induces apoptosis, inhibits the expression of WDR5 targets, and blocks the H3K4me3 enrichment on the promoter of its targets. We also observed the positive correlation of *WDR5* expression with these targets in the cohort study of leukemia patients. Our data reveal that WDR5 may have oncogenic effect and WDR5-mediated H3K4 methylation plays an important role in leukemogenesis.

## INTRODUCTION

Genetic and epigenomic regulation play key roles in hematopoietic homeostasis, the alterations in epigenome and abnormality in enzymatic processes that control epigenomic function are frequently associated with leukemia [1–4]. It is reported that the histone H3Lys4 (H3K4)-methyl epigenome regulates leukemia stem cell oncogenic potential^2^. Mixed-lineage Leukemia (MLL) is a pro-oncoprotein and forms a SET1-like histone methyltransferase complex in leukemia. *MLL* amplification plays an important role in leukemogenesis [3–8]. As leukemia drivers, *MLL* rearrangements result in the fusion of the mixed lineage leukemia gene with other genes, and are one of the most important high-risk leukemia markers [3, 9]. One *MLL*1 allele is truncated and fused in frame with over 70 partners to produce oncogenic *MLL*1 fusion proteins [10–12]; and these fusion proteins stimulate transcription by different mechanisms [13–19]. With rare exception [20], one common feature of the *MLL*1 abnormality in leukemia is the preservation of at least one wild-type *MLL*1 allele with the intact SET domain [21]. This finding suggested that *MLL*1 rearrangement has the limited impact on H3K4me3 level in the leukemic cells, and also H3K4 trimethylation may have more profound effect on oncogenesis in patients with MLL1 rearrangements.

MLL core complex including MLL, WD repeat domain 5 (WDR5), Retinoblastoma-binding protein 5 (RbBP5), and (absent, small, or homeotic)-like (Drosophila) (ASH2) is required for maximal enzymatic activity [22, 23]. WDR5 mediates interaction of the MLL catalytic unit with the core complex and the histone H3 substrate [24]. WDR5 prefers to bind dimethylated H3K4 peptide [25]. WDR5 itself is central for complex assembly and catalyzing H3K4 trimethylation activity [26–28]. These findings indicate that WDR5 is critical for H3K4 methylation. However, the global correlation of *WDR5* with H3K4me3 in leukemia cells has yet been determined.

WDR5 interacts with MLL through the Win motif [29, 30]. Recently it is reported that specifically blocking the MLL1-WDR5 interaction using the inhibitor MM-401 prevents MLL1-WDR5 complex assembly and inhibits MLL1 activity [31]. This inhibitor also blocks proliferation of MLL cells by inducing cell-cycle arrest, apoptosis, and myeloid differentiation; and it induces changes in gene expression similar to those of MLL1 deletion. Similarly, another MLL-WDR5 interaction blocker shows selectively inhibited proliferation and induced differentiation in p30-expressing human AML cells [32]. These reports not only support the key role of MLL1 activity in regulating MLL1-dependent leukemia transcription program but also indicate that WDR5 exerts its role mainly by forming a complex with MLL in leukemia cells.

WDR5 is also reported to be overexpressed in other cancers. WDR5 is hyperexpressed and critical for cell proliferation and H3K4 methylation in human neuroblastoma, prostate cancers and bladder cancers [33–36]. However, very little is known about the role of WDR5 in leukemia, despite our growing knowledge about MLL1 fusion proteins and leukemia. Here, we reported the *WDR5* high expression in human acute leukemia and *WDR5*-mediated H3K4 methylation of oncogenic targets may play an important role in leukemogenesis.

## RESULTS

### Association of WDR5 expression with features of adult ALL

**Figure 1:**
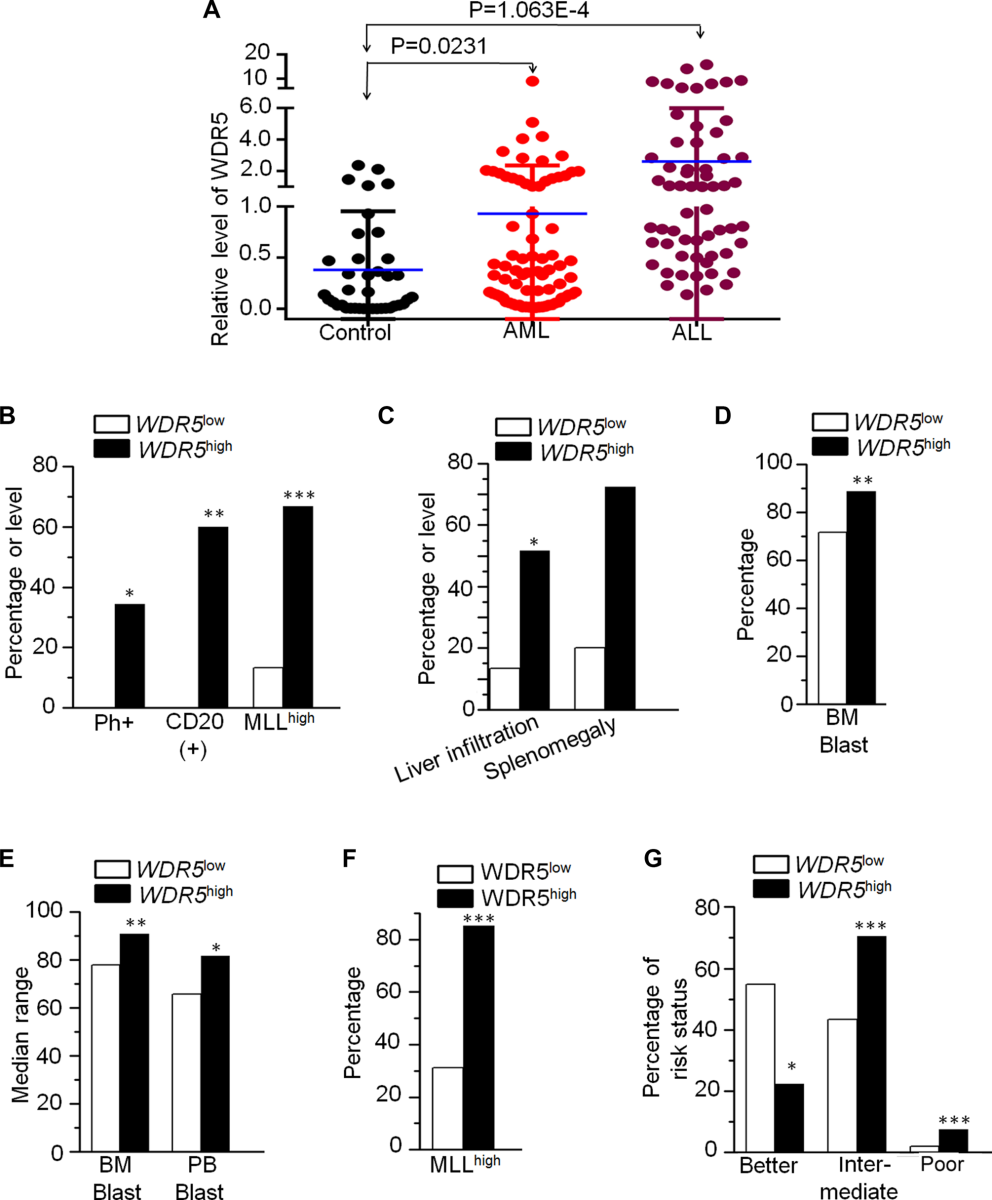
*WDR5* expression in AML and ALL and its correlation with clinical features (**A**) *WDR5* expression in ALL and AML. *WDR5* was detected by qPCR in ALL and AML patients' samples and compared to normal bone marrow tissue (control). The relative level of *WDR5* mRNA was calculated by standard curve. The difference of the *WDR5* expression was analyzed with student *t*-test. *P* < 0.05 are considered as significant. (**B–D**): Correlation of *WDR5* high and low expression with clinical features in patients with ALL. (**E–G**) Correlation of *WDR5* high and low expression with clinical features in patients with AML. *P < 0.05; **P < 0.01; ***P < 0.0001.

We detected *WDR5* mRNA expression in 60 newly diagnosed adult ALL (20 T-ALL and 40 B-ALL) patients, respectively. We found that *WDR5* is significantly more expressed in patients compared to normal controls (Figure 1A), and no significant difference between T-ALL and B-ALL (data not shown). Patients with ALL were divided into high (45 cases) and low (15 cases) *WDR5* expression groups. Patients with high expression of *WDR5* have higher percentage of CD20+ cells (60.0% vs 0.0%, *P* = 0.001), Philadelphia chromosome (Ph) (+)(34.4% vs 0.0%, *P* = 0.026), higher *MLL*1 expressions (66.7% vs 13.3%, *P* = 0.000), splenomegaly and liver infiltration (72.4% vs 20.0%, *P* = 0.001; 51.7% vs 13.3%, *P* = 0.013), and leukemia blast in bone marrow (BM) (87.6% vs 72.4%, *P* = 0.022) compared to patients with low *WDR5* expression (Figure 1B, 1C and 1D; Supplementary Table S1). No significant differences in *WDR5* expression are observed with age, sex, and peripheral blood blasts. These data indicate that high expression of *WDR5* is associated with proliferation and high-risk ALL, suggesting its role in leukemogenesis of ALL.

### Association of *WDR5* expression with characteristics of adult AML

We also detected *WDR5* mRNA expression in 88 newly diagnosed adult AML patients. We found that *WDR5* is significantly higher expressed in AML patients compared to normal control (Figure 1A). Patients were divided into high (27 cases) and low (61 cases) *WDR5* expression groups. Compared to low *WDR5* expression, patients with its high expression showed high median BM blasts (90.8% vs 77.9%, *P* = 0.008) and peripheral blood blast (81.5% vs 66.5%, *P* = 0.049) (Figure 1E, Supplementary Table S2). Similar as ALL, we also observed higher *MLL*1 expression in *WDR5* high expression group (85.2% vs 31.1%, *P* < 0.001) (Figure 1F, Supplementary Table S2). Importantly, when looking at risk status of patients with *WDR5* expression, we found that the favorable-risk is significantly lower in *WDR5* high expression patients (22.2% vs 54.7%, *P* = 0.016), while intermediate-risk and poor-risk are much higher in the high group than that of the low group (70.4% vs 43.4%, *P* < 0.001; 7.4% vs 1.9%, *P* < 0.001). (Figure 1G, Supplementary Table S2). No significant differences in *WDR5* expression were observed with age, sex and WBC. These data indicate that high expression of *WDR5* is associated with high-risk AML, further indicated its oncogenic effect on AML.

### Association of *WDR5* high expression with *MLL*1 high expression in leukemia cells

**Figure 2:**
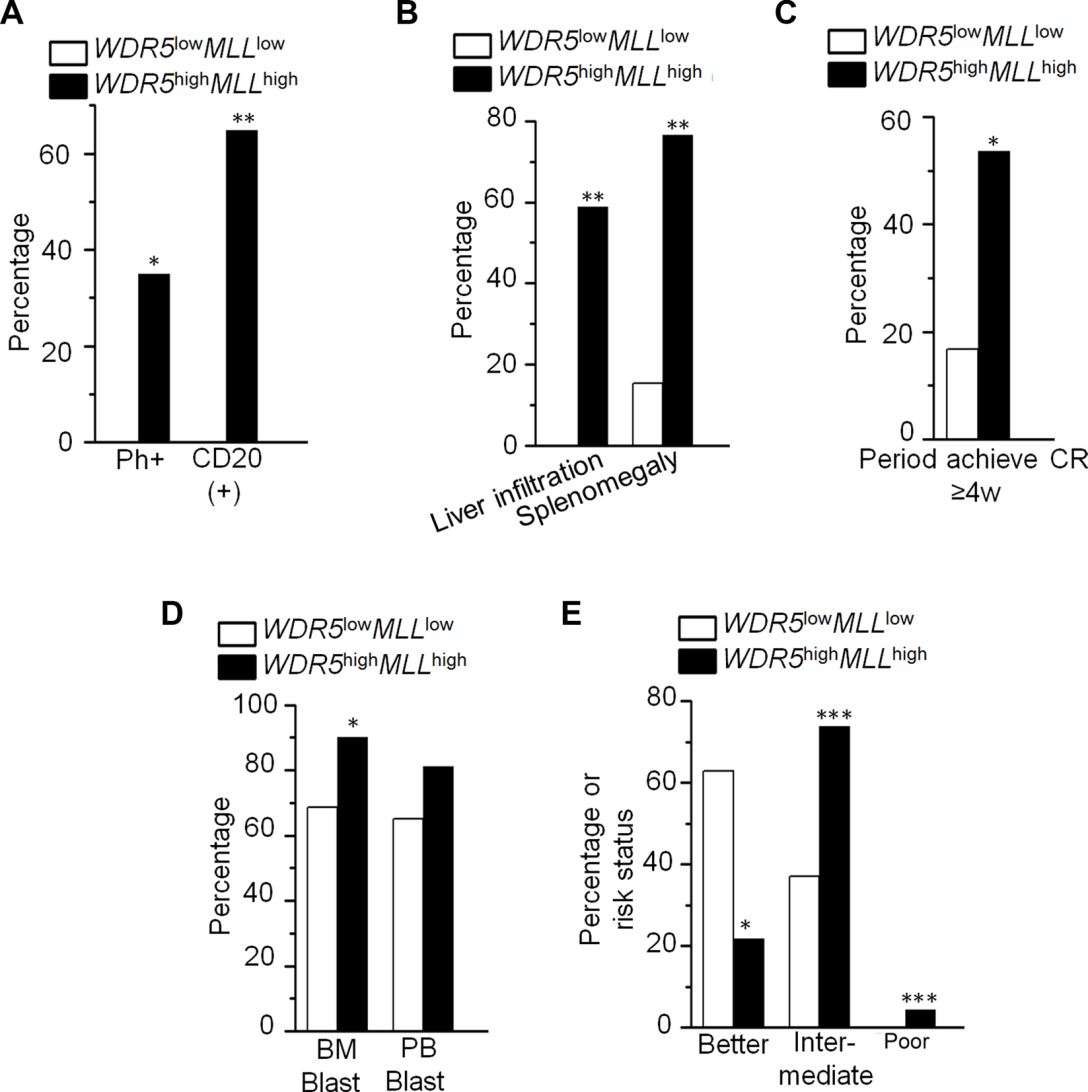
Clinical features of patients with *WDR5*^high^*MLL*^high^ in ALL and AML (**A–C**) Comparison of clinical features of ALL patients with *WDR5*^high^*MLL*^high^ to that with *WDR5* ^low^*MLL*^low^. (**D–E**) Comparison of clinical features in AML patients with *WDR5*^high^*MLL*^high^ to that with *WDR5* ^low^*MLL*^low^. *P < 0.05; **P < 0.01; ***P < 0.0001.

We also analyzed the *MLL*1 expression with *WDR5* expression in ALL and AML patients. We found that high *WDR5* expression is significantly associated with high *MLL*1 expression in ALL and AML patients (Figure 1B and 1F, Supplementary Tables S1 and S2). We also observed that *MLL*1 high expression has higher percentage of CD20+ cells, splenomegaly and liver infiltration compared to patients with low *WDR5* expression in ALL (Supplementary Table S1). *MLL*1 high expression also showed high median bone marrow blasts and significantly higher percentage of intermediate-risk and poor-risk but lower favorable-risk in AML patients (Supplementary Table S2). We further analyzed the clinical features of the patients with both *WDR5* and *MLL*1 high expressions (*WDR5*^high^*MLL*^high^) in the ALL and AML patients. We found that ALL patients with *WDR5*^high^*MLL*^high^ showed higher percentage of Ph+ chromosome (35% vs 0%, *P* = 0.027), CD20(+) cells (64.7% vs 0%, *P* = 0.002), splenomegaly and liver infiltration (76.5% vs 15.4%, *P* = 0.003; 58.8% vs 0%, *P* = 0.001) (Figure 2A and 2B; Supplementary Table S3), and also higher median percentage of BM blasts (87.8% vs 62.0%, *P* = 0.011) than the *WDR5*^low^*MLL*^low^ expression group (Supplementary Table S3). Particularly, the percentage of the period achieving complete remission (CR) more than 4 weeks in the ALL patients with *WDR5*^high^*MLL*^high^ was significantly higher than that of *WDR5*^low^*MLL*^low^ expression (53.6% vs 16.7%, *P* = 0.03) (Figure 2C). AML patients with*WDR5*^high^*MLL*^high^ showed higher median percentage of bone marrow blasts (90.0% vs 68.5%, *P* = 0.004) than the *WDR5*^low^*MLL*1^low^ expression group (Figure 2D, Supplementary Table S3). Based on the cytogenetics of AML patients, the risk status was divided into favorable-risk, intermediate-risk and poor-risk. The risk status analysis data showed that AML patients with *WDR5*^high^*MLL*^high^ had significantly higher percentage of intermediate-risk and poor–risk (73.9% vs 37.1%, *P* < 0.001; 4.3% vs 0.0%, *P* < 0.001) but lower percentage of favorable-risk (21.7% vs 62.9%, *P* = 0.006) (Figure 2E; Supplementary Table S3).

Further, we observed that MLL1 and WDR5 are expressed in leukemia cells (Supplementary Figure S1A). With co-immunoprecipitation assay, we also showed that MLL1 and WDR5 interact in the leukemia cells (Supplementary Figure S1B). These data suggested that WDR5 high expression-induced leukemogenesis may be mediated by interaction with MLL1 in the cells.

### *WDR5* knockdown results in inhibition of cell proliferation and apoptosis

**Figure 3:**
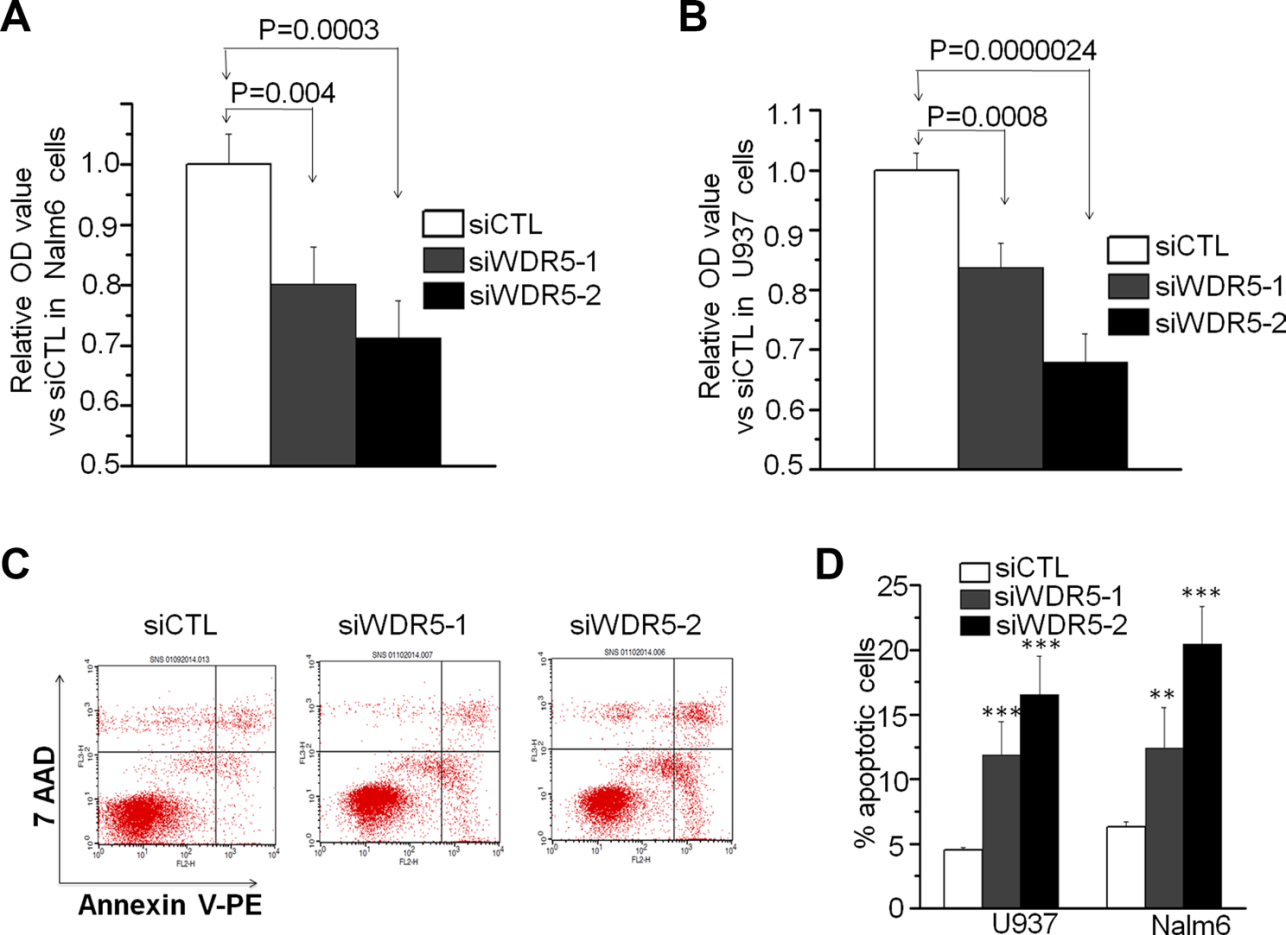
WDR5 knockdown induces proliferation arrest and cell apoptosis in ALL and AML cells (**A–B**) WDR5 knockdown induced the proliferation arrest in Nalm6 B-ALL (A) and U937 AML (B) cells. siWDR5–1=WDR5 shRNA #1, siWDR5–2=WDR5 shRNA #2; siCTL=scramble shRNA. *P* < 0.05 are considered as significant. (**C** and **D**) WDR5 knockdown increased the apoptotic cell rate in the cells. C is the representative flow data in Nalm6 cells. D is the quantitation data in Nalm6 and U937 cells. **P < 0.01; ***P < 0.0001.

Next, we explored the effect of *WDR5* knockdown in cell growth of leukemia cells. With WST-1 cell proliferation assay, we found that *WDR5* knockdown with shRNA significantly suppress the proliferation of Nalm6 and U937 cells compared to that of scramble shRNA control (siCTL) (Figure 3A and 3B). We also observed the effect of *WDR5* knockdown on apoptosis of the cells. Figure 3C is the flow cytometry chart for the apoptosis assay in Nalm6 cells. The quantitative data showed that *WDR5* shRNA significantly increases the apoptosis of Nalm6 and U937 cells compared to control (Figure 3D). These data revealed that *WDR5* is essential for the cell growth of the leukemia cells, further suggesting the oncogenic role of *WDR5* in leukemia.

### Genome-wide targeting of WDR5 in leukemia cells

We determined the genome-wide occupancy of WDR5 and using chromatin immunoprecipitation followed by deep-sequencing (ChIP-seq) in human RS4;11 ALL and THP-1 AML leukemia cells, we identified 1490 and 515 target genes for WDR5 in RS4;11 and THP-1 cells, respectively. ChIP-seq data for WDR5 were validated by quantitative chromatin immunoprecipitation (qChIP) analysis of the high and low rank ChIP-seq peak values (data not shown).

**Figure 4:**
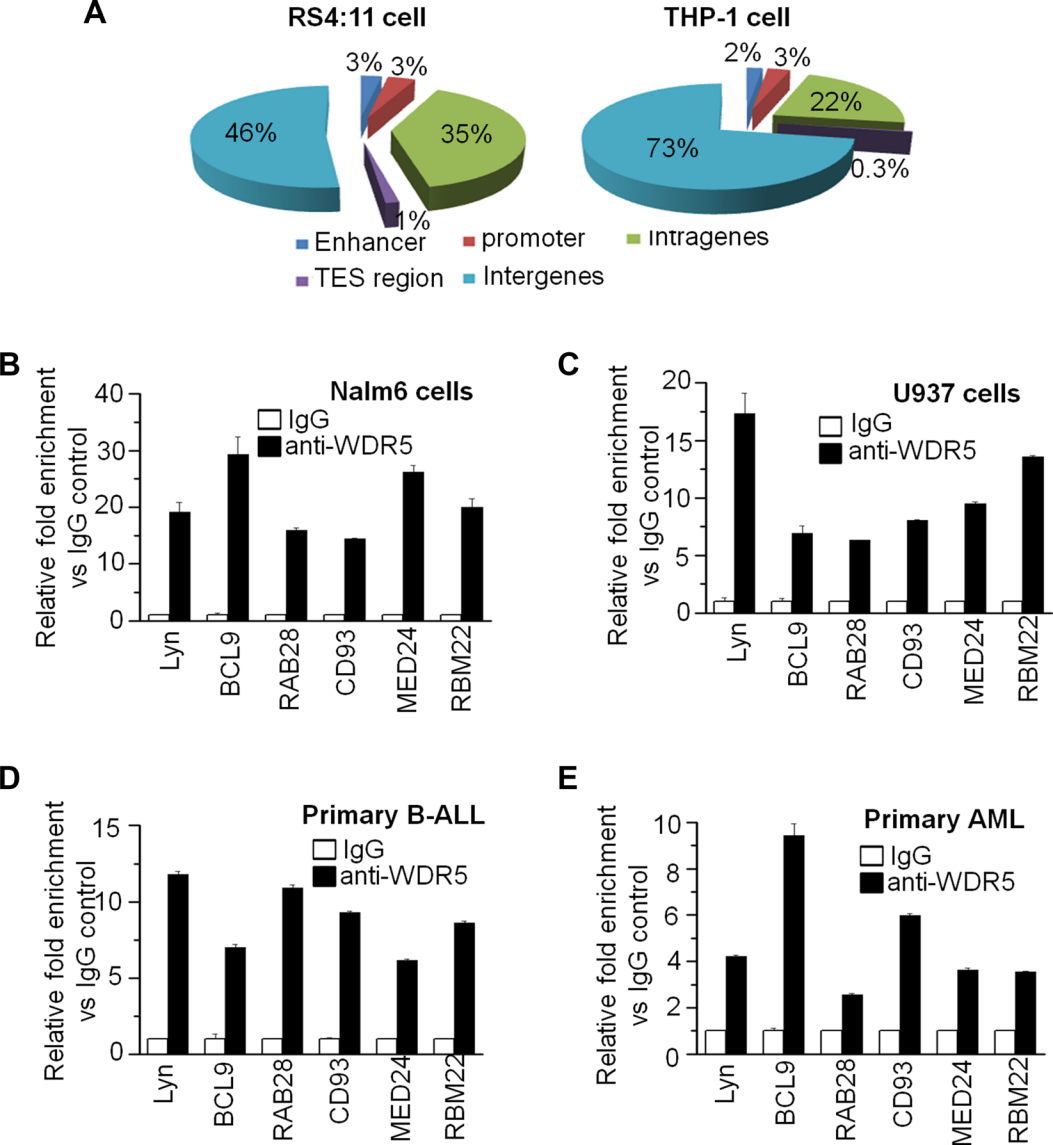
Genome-wide distribution of WDR5 and Gene Ontology (GO) analysis (**A**) Percentages of peaks that bind sites in each location are given. (**B–E)** qChIP data showed that WDR5 binds to the promoter region of its targets identified by ChIP-seq in leukemic cell lines-Nalm6 (B) and U937 cells (C) as well as primary B-ALL (D) and AML (E).

Analysis of the distribution of WDR5 peaks in genomic regions showed that more than half of WDR5 peaks are located in intergene regions and about 5–6% of peaks are in promoter and enhancer regions (Figure 4A). We also did the analysis of GO term and pathways in the gene targets, and found the WDR5 binding genes are involved in multi-oncogenic signaling, apoptosis, transcriptional regulation, histone modification, cell proliferation and apoptosis, cell adhesion and metabolism, etc. (Supplementary Figure S1C).

We further observed WDR5 binding on its targets in ALL and AML cell lines by qChIP. WDR5 significantly binds to promoter region of Tyrosine-protein kinase Lyn (Lyn), B-cell CLL/lymphoma 9 (BCL9), Ras-related protein Rab-28 (RAb28), Cluster of Differentiation 93 (CD93), Mediater complex subunit 24 (MED24) and RBM22 in Nalm6 B-ALL cells (Figure 4B) and U937 AML cells (Figure 4C). Importantly, WDR5 also significantly binds to these target genes in primary B-ALL (Figure 4D) and AML (Figure 4E) cells.

### Global association of WDR5 peaks and H3K4me3 peaks in leukemia cells

**Figure 5:**
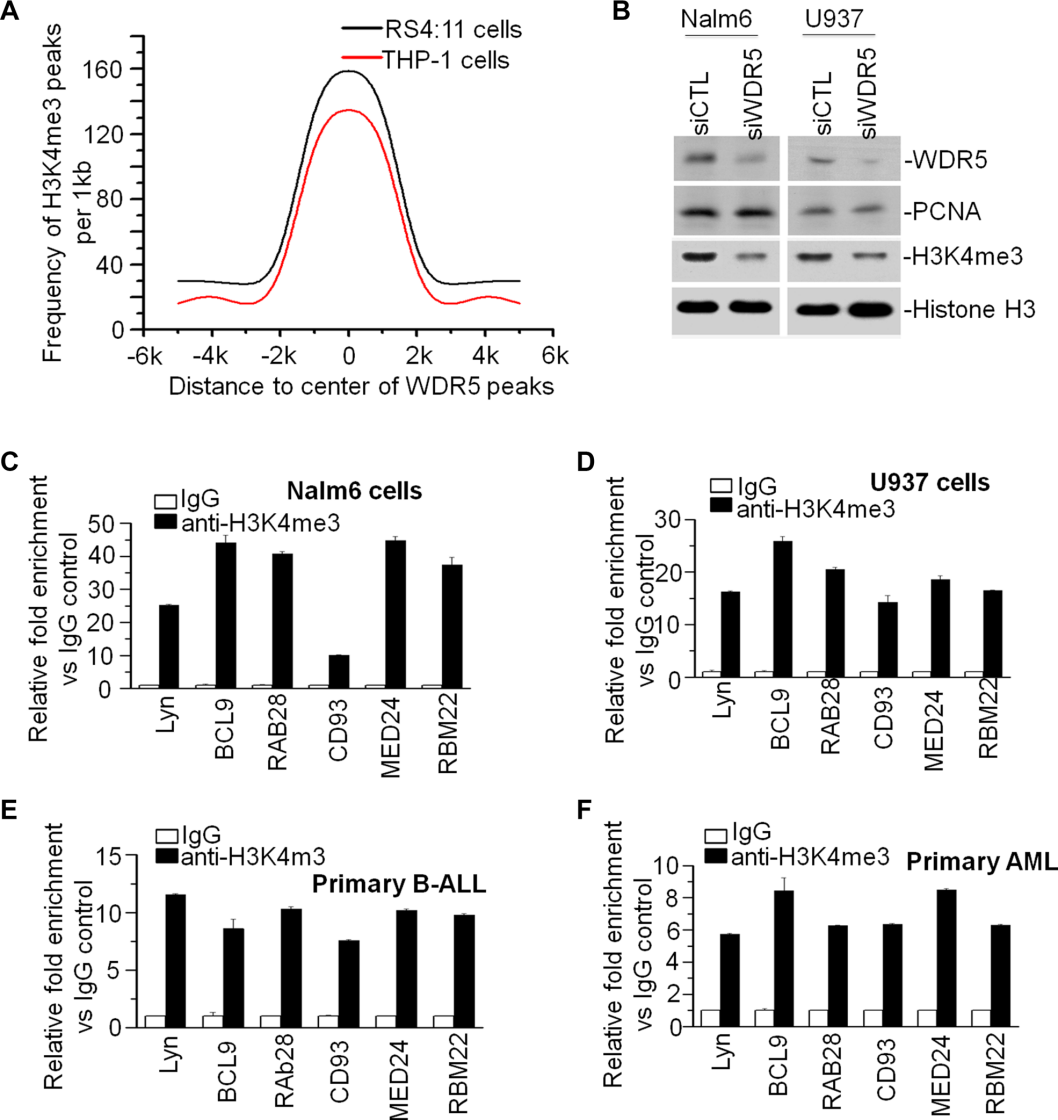
WDR5 associates with MLL in leukemia cells and global accumulation of H3K4me3 around the WDR5 binding peaks (**A**) Distribution of H3K4me3 peaks relative to the center of WDR5 peak in RS4;11 and THP-1 cells. Graphed is the frequency of H3K4me3 in all WDR5 peaks, over a 1 kb span. (**B**) Effect of WDR5 knockdown on H3K4me3 level. (**C–F**) H3K4me3 enrichment in the promoter region of WDR5 targets in cell lines, Nalm6 (C) and U937 (D) cells, and primary B-ALL (E) and AML (F) cells.

It is reported that the inhibitors blocking WDR5-MLL1 interaction affects the global H3K4me3 level in leukemic cells [31, 32], however it has no report about global correlation of WDR5 binding with H3K4me3 binding targets. To provide insights into the epigenetic mechanisms that determine expression of WDR5 target genes in the cells, we identified the H3K4me3 binding profiling in RS4;11 and THP-1 cells by ChIP-seq. The distribution of H3K4me3 peaks in genomic regions is showed on Supplementary Figure S2. We also analyzed the distribution of H3K4me3 peaks relative to WDR5 peaks and found that WDR5 peaks are enriched with the presence of H3K4me3 peaks (Figure 5A). We further observed the obvious reduction of H3K4me3 level in both Nalm6 and U937 cells upon WDR5 knockdown compared to that in scramble shRNA control (Figure 5B). These data indicated that WDR5 not only associates with H3K4me3 but also affects H3K4me3 generation in the cells. We also observed that WDR5 and H3K4me3 peaks are simultaneously presented in the promoter region of WDR5 targets using genome browser (Supplementary Figure S3). These data indicate that WDR5 is essential for the H3K4 methylation on the promoter of its targets. Then, we further observed the enrichment of H3K4me3 in the promoter region of WDR5 targets and found that H3K4me3 is dramatically enriched in the promoter region of Lyn, BCL9, RAB28, CD93, MED24, and RBM22 in Nalm6 (Figure 5C) and U937 (Figure 5D) cells as well as primary B-ALL (Figure 5E) and AML (Figure 5F) cells.

### WDR5 controls the expression of its targets

**Figure 6:**
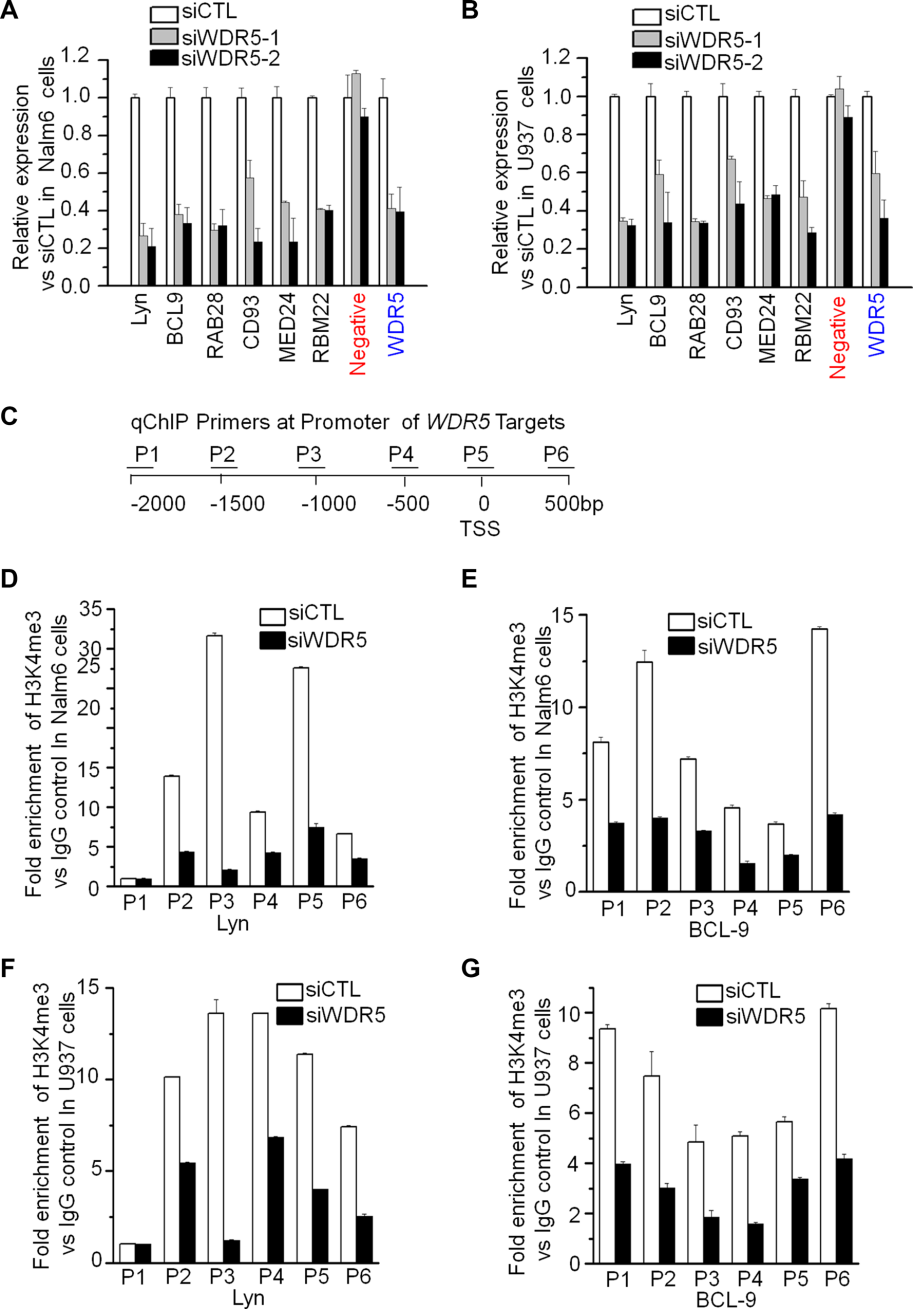
WDR knockdown suppresses the expression of its targets and H3K4me3 binding to the promoter of its targets (**A–B**) The expression of WDR5 targets inn Nalm6 (A) and U937 (B) cells treated with siWDR5. Cells treated with siWDR-1 or −2 shRNA and scramble shRNA (siCTL) for two days, total RNA was isolated for gene expression by qPCR. (**C**) Location of the primers for PCR of promoter of Lyn and BCL9. (**D–F**) H3K4me3 binding on Lyn and BCL9 in Nalm6 (D, E) and U937 (**F**, **G**) cells. Cells treated with siWDR shRNA and siCTL for two days, qChIP assay was performed for the binding with anti-H3K4me3 antibody.

We analyzed the correlation of *WDR5* expression with that of its targets in the cohort study of ALL and AML patients, and observed a positive correlation of *WDR5* expression with that of Lyn (Supplementary Figure S4A and S4B), MED24 (Supplementary Figure S4C and S4D), RBM22 (Supplementary Figure S4E and S4F), which are important for oncogenesis and apoptosis. To further assess the effect of WDR5 on expression of the target genes, Nalm6 and U937 cells were expressed with WDR5 shRNA or control scramble shRNA. Reduced WDR5 expression resulted in reduced transcription of WDR5 target genes—Lyn, BCL-9, RAB28, CD93, MED24 and RBM22 (Figure 6A and 6B). Reduced expression of Lyn and BCL9 was also observed in the WDR5 knockdown RS4;11 and THP-1 cells (Supplementary Figure S5A and S5B). Moreover, WDR5 knockdown obviously reduced the WDR5 binding to the promoter of its targets, Lyn, BCL9 and RBM22 (Supplementary Figure S6). This data indicate that WDR5 is responsible for transcription of these genes and suggest that WDR5 promotes leukemia via transcriptional activation of genes.

### WDR5 is essential for H3K4me3 enrichment in the promoter of its targets

We further analyzed the WDR5 dependence of H3K4me3 enrichment in the promoter of its targets. WDR5 was knocked-down by shRNA in Nalm6 and U937 cells. The enrichment of H3K4me3 in the promoter region of Lyn and BCL9 was determined by qChIP. Figure 6C showed the location of the primers that we used for PCR of the genes in the ChIP'd DNAs. WDR5 knockdown blocks the H3K4me3 enrichment in the promoter region of WDR5 targets –Lyn and BCL9 (Figure 6D–6G). Similar results are observed in MLL-rearranged RS4;11 and THP-1 cells (Supplementary Figure S5C and S5D). These data indicate that WDR5 is essential for histone 3 lysine 4 methylation in the promoter region of its targets, and also suggest that WDR5 activates gene expression via increasing H3K4me3 levels of the genes.

## DISCUSSION

There is no report about WDR5 expression and its correlation with clinical features in leukemia.

Our data first showed the WDR5 high expression particularly the WDR5^high^+MLL1^high^ expression in leukemia patients and their correlations with high-risk ALL and AML leukemia; and revealed the oncogenic effects of high *WDR5* expression in acute leukemia by activation of its oncogenic targets via epigenomic regulation, as shown on a model of Supplementary Figure S7.

### Oncogenic effect of WDR5 high expression

The crystal structural analysis suggests that WDR5 has many exposed surfaces which make it a perfect adaptor to interact with other proteins. It has been shown that WDR5 prefers to bind dimethylated histone H3Lys4 peptide [25]. We observed the co-localization of WDR5 binding with H3K4me3 globally. WDR5 knockdown results in the cell proliferation arrest and apoptosis in leukemic cells, and induces suppression of expression of its targets and loss of H3K4me3 enrichment in the promoter region of the targets. Clinical cohort data also showed that *WDR5* expression is positively correlated with the oncogene Lyn, RAB28 (data not shown), and anti-apoptotic factor BCL9. These data reveal that WDR5 may have an oncogenic effect in leukemia.

Moreover, WDR5 functions as a common subunit of all six mammalian histone H3Lys4 methyltransferases [37, 38]. MLL1 protein also interacts with WDR5 by Win motif next to the SET domain [29, 30]. We also found that WDR5 is associated with MLL1 in the leukemia cells. WDR5 knockdown induces the reduction of H3K4me3 level. Clinical data showed that ALL and AML patients with *WDR5*^high^*MLL*^high^ have high-risk leukemia or are in high-risk status. These data indicate that WDR5 could exert its oncogenic effect mainly by recruitment of MLL1 to increase H3K4me3 levels in the promoter region of its targets. This would further result in the activation of oncogenic signaling, even in *MLL* rearrangement patients, although it is also reported that WDR5 itself is central for complex assembly and catalyzing H3K4 trimethylation activity [26–28].

### A novel oncogenic mechanism in ALL and AML with MLL rearrangement

MLL rearrangement occurs in approximately 10% of human acute leukemia, and are present in > 70% of infant ALL cases, 35–50% of infant AML cases and secondary or therapy-related leukemia [3, 9]. In the most common MLL1 rearrangements, the MLL1 allele is truncated and fused in frame with over 70 partners to produce oncogenic MLL1 fusion proteins (e.g., MLL-AF9, MLL-ENL) [10–12]. Previous work has shown that some MLL1 fusion proteins, especially those forming the EAP complex [13], are recruited to Hoxa9 and directly stimulate transcription elongation by recruiting cofactor complexes such as PAF1C [14, 15], DOT1L [16–18], and pTEFb/BRD4 [19]. We observed many H3K4me3 binding sites in the MLL1 rearrangement cell lines, and also showed that *WDR5* globally co-localizes with H3K4me3. WDR5 knockdown results in a significant decrease of H3K4me3 enrichment in the promoter region of its targets in both the cells without MLL rearrangement (Nalm6 and U937 cells) as well as that with MLl1 rearrangement (RS4;11 and THP-1 cells). We also observed that *WDR5* is highly expressed in acute ALL and AML, and high expression of both *WDR5* and *MLL* are correlated with high risk-ALL and -AML. Our findings revealed that high expression of WDR5, a key member of the MLL complex, is the novel mechanism responsible for oncogenesis in ALL and AML by increasing Histone H3 Lys4 methylation on its oncogenic gene targets in patients with or without MLL rearrangement. High expression of*WDR5* results in the enrichment of H3K4me3 in the promoter region of its targets by increasing recruitment of MLL1 methyltransferase. In cells with MLL1 rearrangement, high expression of *WDR5* induces the enrichment of H3K4me3 on its targets by recruiting more wild type MLL1 and through the methyltransferase activity of WDR5 itself.

### WDR5 targets responsible for its oncogenic effect

It is reported that WDR5 drives the expression of protumorigenic gene-MDM2 with c-MYC in neuroblastoma [35]; WDR5 high expression promotes proliferation, self-renewal and chemoresistance in bladder cancer via mediating H3K4 trimethylation on cell cycle genes such as CCNA, CCNB1, CCND1, CCNE1 etc. [36]; Also it has reports that blocking of MLL-WDR5 interaction and MLL1 knockdown induced the suppression of c-MYC and BCL-2 in leukemia cells [27]; and loss of WDR5 increases expression of G-CSF, ccl9, etc., and restores granulocytic differentiation potential in C/EBPa p30-expressing AML cells [32]. However, it has no report about the WDR5 global binding gene targets in leukemia cells. Here, we identified around 2000 WDR5 binding genes, and GO term analysis showed that these genes are involved in biological functions such as transcriptional regulation, chromatin remodeling, multi-oncogenic pathways, apoptosis regulation, metabolism, cell adhesion, and etc.

Lyn, a gene target of WDR5, is a tyrosine kinase belonging to the Src family kinases (SFKs) and plays a crucial role in the onset and progression of CLL [39–42]. It has been reported that the existence of a TK network composed of the tyrosine AXL, Lyn and SYK, promotes cell resistance to nilotinib treatment and drives Bcr–Abl-independent CML cell proliferation. The up-regulation of Lyn and other TKs is observed in TKI-resistant CML cells [34, 43] and high Lyn expression in leukemia [41, 44]. Lyn plays a critical role in oncogenesis of AML [45]. Lyn is also responsible for anti-apoptosis in the cancer cells [46]. We observed the positive correlation of *WDR5* expression with Lyn in the cohort study of ALL and AML patients, and also *WDR5* high expression significantly appeared in Ph(+) patients. *WDR5* knockdown induced the down-regulation of Lyn, indicating *WDR5*-mediated transcriptional activation of Lyn may play important roles in oncogenesis as a downstream target of *WDR5* high expression in acute ALL and AML.

Other WDR5 targets, such as BCL9, the anti-apoptosis BCL-2 family member, is a proto-oncogene previously characterized as co-activator of Wnt/ß-catenin signaling, for mammary tumorigenesis in mice and humans [47]. WDR5 target RAB28, member of the Ras oncogene family, is reported to be differentially expressed in swine melanoma [48]. Another WDR5 target, MED24 is a component of the mediator complex, a transcriptional coactivator complex requires for the expression of almost all genes. It is reported that MED24 is overexpressed in breast cancer cells [49]. *WDR5* target RBM22 is an RNA binding protein that may play a role in cell division and may be involved in pre-mRNA splicing. We found that *WDR5* expression is positively correlated with expression of BCL9, MED24 and RAB28 in the cohort study of ALL and AML patients. Notably, WDR5 knockdown dramatically suppress expression of these targets in the leukemia cells. These data revealed that high expression of *WDR5* is critical for transcriptional activation of these targets, which may be further responsible for the onset of oncogenesis and cell proliferation in leukemia. Moreover, WDR5 is reported to be involved in cell differentiation and reprogram of stem cells [57]; we did observe some WDR5 targets involving cell differentiation, therefore the effect of WDR5 knockdown on differentiation of the ALL and AML cells will be further clarified.

In summary, we identified that *WDR5* is highly expressed in adult ALL and AML, and showed its correlation with high-risk leukemia. We also identified the WDR5 binding profile in leukemia cells and found WDR5 bindings globally co-localized with H3K4me3 in leukemia cells. Our finding revealed the WDR5 knockdown results in the proliferation arrest and apoptosis of leukemia cells, and also decreased the expression of its targets through loss of H3K4me3. Our data reveal that WDR5 has oncogenic effects and WDR5-mediated H3K4 methylation may play an important role in leukemogenesis.

## MATERIALS AND METHODS

### Patients and samples

The 151 patients' BM samples [90 male, 61 female; median age 32 (14–77) years old] with AML (91 patients) and ALL (40 B-ALL, 20 T-ALL), and the 30 volunteers' BM samples for control were collected between June 2008 and June 2014 at the First Affiliated Hospital of Nanjing Medical University. The ALL and AML diagnosis was made according to the cytogenetic, morphologic, Immunophenotypic and molecular criteria of WHO Diagnosis and Classification of ALL (2008). The written informed consent was provided by all patients and volunteers in accordance with the Declaration of Helsinki before enrollment in the study. The cohort study was also approved by the Institutional Review Board of the Nanjing Medical University. Cytogenetic and molecular analyses as previously reported.

### Cytogenetic and molecular analyses

Conventional cytogenetic analysis was performed at the time of diagnosis by using unstimulated short-term cultures according to the recommendations of the International System for Human Cytogenetic Nomenclature (ISCN). At least 20 BM metaphase cells were analyzed for each sample.

The flow cytometry was performed to do immunophenotypic analyses on fresh pretreatment BM samples. The cell-surface antigen was defined as positive when fluorescence intensity of at least 20% of cells exceeded fluorescence of negative control as previously described [45, 46].

Risk status in AML patients was classified by cytogenetics and molecular abnormalities with National Comprehensive Cancer Network (NCCN) Guideline Version1–2015. Favorable-risk: inv(16) or t(16;16); t(8;21); t(15;17); normal cytogenetics with NPM1 mutation or isolated CEBPA mutation in the absence of FLT3. Intermediate-risk: Normal cytogenetics; +8; t(9;11); Other non-defined; t(8;21), inv (16), t(16;16) with c-KIT mutation. Poor-risk: Complex (3 abnormal clones); −5, 5q−, −7, 7q−; 11q23 - non t(9;11); inv(3), t(3;3); t(6;9); t(9;22); Normal cytogenetics with FLT3-ITD mutation.

### Cell culture, reagents, plasmid construction, retroviral gene transfer

RS4;11, U937, THP-1 and Nalm6 cells were obtained from American Type Culture Collection (ATCC, Manassas, VA) and cultured in RPMI 1640 medium (Cellgro) supplemented with 10% fetal bovine serum (Hyclone). Cells were incubated at 37°C in a humidified atmosphere of 5% CO_2_.

Primary leukemia cells were obtained from the Anonymous patient with B-ALL and AML at First Affiliated Hospital of Nanjing Medical University, in compliance with Institutional Review Board regulations.

### ChIP-seq assay

ChIP-seq assays for WDR5 and H3K4me3 in leukemia cells were performed as previously reported [50–53] and as described for qChIP assay. For ChIP-seq library, cells were cross-linked for 10 min in PBS containing 1% formaldehyde and reaction was stopped by adding glycine. Cell pellets were flash frozen and stored at −80°C. Chromatin was fragmented using the Bioruptor sonicator (Diagenode) for 30 min (30s pulses, 30s pauses in between) (for WDR5) or using Micrococcal nuclease (for H3K4me3) to produce fragments ~400 bp in size. ChIP assays were performed by incubation of the chromatin with anti-WDR5 antibodies (ab56919) or H3K4me3 antibody (ab8580), which was pre-coated onto Goat-anti-rabbit IgG Dyneabeads (Invitrogen). Following overnight incubation at 4°C, protein/DNA complexes were extensively washed and eluted. The ChIPed DNA was de-crosslinked, digested with proteinase K, extracted with phenol/chloroform, and then treated with RNaseA. Finally, the DNA was purified using the QIAquick PCR Purification kit (QIAGEN). ChIP-seq libraries were created using ChIP- seq DNA sample prep kit (Illumina), size-selected and the 200– 400 bp fraction was extracted and purified. Libraries were sequenced at Genomics Center BTG company (Beijing, China). Sequence fastq files were aligned to the UCSC human genome assembly HG19 using the Eland application, allowing no more than two mismatches per sequence. Only sequences aligning uniquely to the human genome were used to identify peaks. Peaks were called using MACS with default parameters.

ChIP-Seq data are accessible on GEO with an access number of http://www.ncbi.nlm.nih.gov/geo/query/acc.cgi?token=ibghmioodfqdlqx&acc=GSE72864.

### Bioinformatic analysis of ChIP-seq data

Annotation of the peaks, distribution of peaks in different gene regulatory regionsand relation of WDR5 peaks and H3K4me3 peaks are analyzed as our previous reports [52, 53].

### Annotation of the peaks

Peak annotation with associated genes was retrieved with a Perl script. In this script program, the peak information was input from the MCAS output peak files in “COD” format. Then, the nearest gene to each peak and associated locations (gene start, gene end, 5′UTR, 3′UTR, exon and intron) in the human genome was identified using the built-in program. The gene names from HGNC [54] were also input and passed. The output data file includes the integrated information of peaks, genes and annotations.

### Distribution of peaks in different gene regulatory regions

The number of peaks in different gene regulatory regions (e.g. promoter and intergenic regions) was calculated using a Perl script and then the distribution of the peaks in each treatment was depicted in a Figure in a column chart or a pie graph using Microsoft Excel. The peaks passing each position in relation to TSS (Transcriptional Start Site) and TES (Transcriptional End Site) was also counted by a Perl script program. In this program, the maximum number of peaks at a position was also recorded. The percentage of the number of peaks passing each position over the maximum number of peaks at a position was computed and then output into a tab-delimited text file. Finally, the output data was plotted using the Microsoft Excel or Origin software package.

### Relation of WDR5 peaks and H3K4me3 peaks

The H3K4me3 peaks were mapped onto the WDR5 peaks at distances of 50 bp, 100 bp, 200 bp, 500 bp, 1 kb, 2 kb, 3 kb, 4 kb and 5 kb from the centers of the H3K4me3 peaks to the center of the WDR5 peak using a Perl script program.

The H3K4me3 densities per 100 WDR5 peak/kb were calculated using the equation:

HMd = (Y_i_ − Y_i-1_)*100/[(X_i_–X_i-1_)*M]

Where:

HMd represents H3K4me3 density

Y_i_ is the peak number of H3K4me3 i.

X_i_ is studying distance from a H3k4me3 peak center to a WDR5 peak center

M is the peak number of WDR5 peaks.

The resulting data were plotted into graphs using the Original software package.

### Quantitative chromatin immunoprecipitation (qChIP)

The qChIP assay was done as previous reported [52, 53]. Briefly, 1 × 10^7^ cells were collected and cross-linked in cross-link solution containing 1% formaldehyde for 10 min on ice. The reaction was stopped by adding glycine to a final concentration of 0.125 M. The ChIP samples were prepared as follows: cells per condition were treated with solution I (50 mM Hepes KOH, pH 7.5, 140 mM NaCl, 1 mM EDTA, 10% Glycerol, 0.5% NP-40, 0.25% Triton X-100, protease inhibitor) with rotation at 4°C for 10 min. The pelleted cells further treated with solution II 0.2 M NaCl, 1 mM EDTA pH 8.0, 0.5 mM EGTA pH 8.0, 10 mM Tris pH 8.0, protease inhibitor) with rotation for 10 min at room temperature. The chromatin was fragmented in solution III (1 mM EDTA pH 8.0, 0.5 mM EGTA pH 8.0, 10 mM Tris pH 8.0, protease inhibitor) with a Bioruptor (Diagenode,) for 30 min (30 s pulses, 90 s pauses) to obtain an average size of 400 bp and the chromatin was centrifuged at 4000 RPM for 10 min at 4°C and added with 10% glycerol. The WDR5 (ab56919) and H3K4me3 (ab8580) antibodies and normal rabbit IgG (ab171870) as control [53] pre-coated onto Goat-anti-rabbit IgG Dyneabeads (Invitrogen) are used and incubated with chromatin overnight at cold room. Protein/DNA complexes were captured with a Magnetic Particle Concentrator (Invitrogen). Crosslinks were reversed. Samples were treated with proteinase K and RNaseA. DNA was then recovered using the QIAquick PCR Purification kit (QIAGEN). Enrichment of the ChIP sample over input was evaluated by qPCR with three or more replicates, using specific primers in the promoter region of WDR5 target (Supplementary Table S4). Relative concentration of the qPCR product was presented as the fold change of the level of DNA-*WDR5* samples in comparison to controls.

### Real Time-PCR

Total RNA was isolated using the RNeasy Mini Kit (QIAGEN). A 1 μg aliquot of RNA was reverse transcribed using a SuperScript^™^ First-Strand Synthesis System for RT-PCR Kit (Invitrogen). qRT-PCR was performed with qSTAR SYBR Master Mix (OriGene) using a StepOne Plus real-time PCR system (Applied Biosystems). Each experiment was performed in triplicate.

In order to quantitate gene expression value in patients' samples, template standards and primers against *Homo sapiens* gene *WDR5* (HK204310, OriGene, USA) and *Homo sapiens* housekeeping gene *GAPDH*, HK203273, OriGene, USA) were obtained from OriGene Technologies (Rockville, MD). Gene expression values of the genes of interest (GOI) were achieved in each patient by a formula obtained with a scatter graph of the Ct values from the serial dilutions of template standard following manufacturer's instruction and as previously reported [40, 44]. The expression level of GOI was subsequently normalized to the housekeeping gene, expressed as gene expression value of GOI/GAPDH.

All the patients were divided into high and low *WDR5* expression groups (Q3–4 vs Q1–2) and the cutoffwas determined by SPSS 17.0. Statistical analysis with analysis of variance (ANOVA) showed that the difference of *WDR5* expression in the two groups was very much significant (*P* < 0.0001).

The qPCR for expression of WDR5 targets in the WDR5 knockdown cells was performed and the results were normalized to those obtained with 18sRNA and presented as fold induction over vector controls. Primers for the qPCR are listed in Supplementary Table S4. Primer for *18s RNA*, Sense: 5′-GTAACCCGTTGAACCCCATT-3′, Antisense: 5′-CCATCCAATCGGTAGTAGCG-3′.

### Co-Immunoprecipitation

THP-1 cells were homogenized in radioimmune precipitation assay buffer containing 25 mM Tris-HCl (pH 7.4), 150 mM NaCl, 60 mM n-octyl-D-glucoside, 1% Triton X-100, 2 mM PMSF, and a protease inhibitor tablet/50 ml. The homogenate was centrifuged to remove insoluble debris. 400 μg of supernatant was used for each reaction and was precleared with protein A/G-Sepharose beads (Pierce). The precleared supernatant was incubated in primary antibody overnight at 4°C, and protein-antibody complexes were recovered using recombinant protein A/G-Sepharose (Pierce). As a negative control, isotype control IgG was used. Cell lysates were separated on precast 10–20% SDS-PAGE gels and immunoblotted as described previously [46].

### WDR5 shRNA knockdown and Western blot

The Nalm6 and U937 cells were transiently transfected with human *WDR5* shRNA constructs in the GFP vector (pGFP-v-RS) (Origene) using the Neon Transfection System (Invitrogen). The 29-mer scrambled shRNA cassette in the pGFP-VRS vector was also used as a control. After transfection for 2 days, cells with transfection efficiency above ~80% and cell viability greater than 95%, were harvested for cell proliferation and apoptosis assay, and also for total RNA isolation. Knockdown of *WDR5* was confirmed by measurement of *WDR5* mRNA level with qPCR. Primers: *WDR5*-F: 5′-CC GAACGGCAAATACATCCT-3′, *WDR5*-R: 5′-TGTA GTCCCAGAGCTTCAGAGTGT-3′.

WDR5 protein level was also determined by western blot with anti-WDR5 antibody in the knockdown cells and scramble shRNA control. PCNA protein level was also examined as loading control.

### Histone extraction

Histone extraction was performed using the Abcam histone extraction protocol (www.abcam.com). The protein was quantified using the Bradford assay and used for western blot analysis.

### Proliferation and cytotoxicity assays

The colorimetric WST-1 cell proliferation assay (Roche Applied Science; 11644807001) was performed in 96-well white clear bottom plates (Costar, 3610) in quadruplicate, according to manufacturer's instructions [52, 54]. Absorbance at 440 nm (reflects number of viable cells) was measured using a BioTek Synergy Mx plate reader. Cytotoxicity assays were performed in triplicpate by incubating 1.0 × 10^6^ cells per well 24-well plate in the presence or absence of drugs in a final volume of 1 ml. Aliquots of cells were harvested at indicated time periods and hemocytometer count using Trypan blue exclusion was used to obtain viable cell counts.

### Determination of apoptosis by flow cytometry

Apoptosis was measured by staining cells with PE Annexin V and 7-AAD [49, 52]. Cells were analyzed and quantified by flow cytometry (MODFit). Nalm6 and U937 cells were transfected with WDR5 shRNA or scramble shRNA as control, respectively. Two days after the transfection, the cells were harvested, washed and then stained with fluorochrome-conjugated Annexin V following the manufactory's Manual (Bioscience). Annexin V and 7-AAD negative were identified as live cells, whereas 7-AAD positive alone were dead cells. Cells which were positive for Annexin V but were 7-AAD negative were identified as early apoptosis. Cells which were both Annexin-V and 7-AAD positive were identified as cells in late apoptosis. The ratio of the apoptotic cells included both early and late apoptotic cells.

### Statistical analysis

Patients were divided into high and low *WDR5* expression groups (Q3–4 vs Q1–2) [55, 56]. For quantitative parameters, overall differences between the cohorts were evaluated using a Mann – Whitney *U*-test. For qualitative parameters, overall group differences were analyzed using a χ^2^ test. All statistical analyses were performed using the SPSS 17.0 and *P* < 0.05 was considered statistically significant.

The experimental data are shown as the mean value with bars representing the standard error of the mean (S.E.M.). Determinations of statistical significance were performed using a Student *t*-test for comparisons of two groups or using analysis of variance (ANOVA) for comparing multiple groups. The *P* < 0.05 was considered statistically significant. All data fit for normal distribution with *t* or *u* test, and the data for ANOVA analysis are homogeneity data

## SUPPLEMENTARY MATERIALS FIGURES AND TABLES


