# Supplementary material for: WDR5 high expression and its effect on tumorigenesis in leukemia
Source: Oncotarget. 2016 May 12;7(25):37740–54. doi: 10.18632/oncotarget.9312 (PMC5122345; doi:10.18632/oncotarget.9312)
Supplement: Supplementary file 1 [file oncotarget-07-37740-s001.pdf]

WDR5 high expression and its effect on tumorigenesis in leukemia

Supplementary Materials

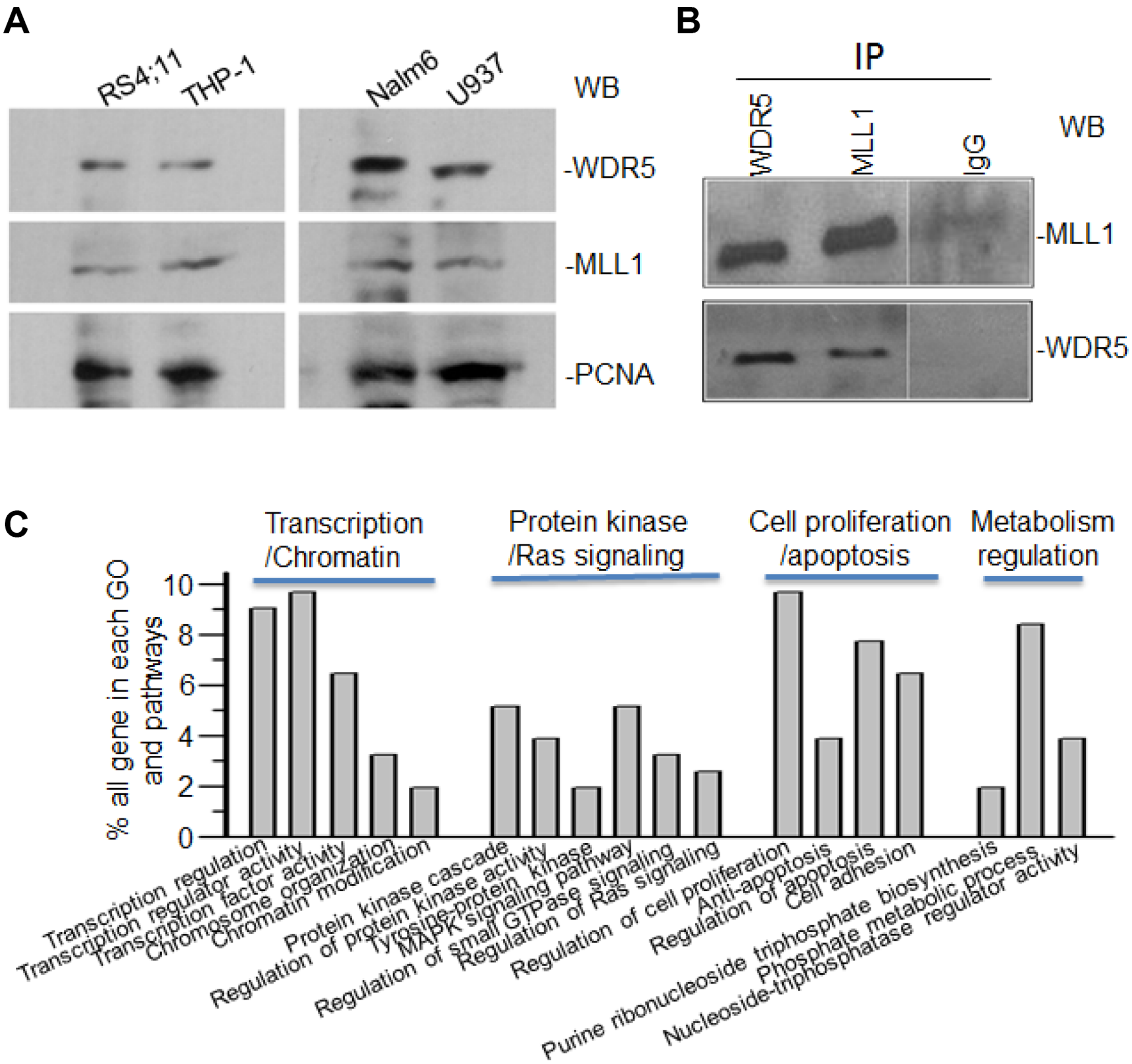

Supplementary Figure S1: WDR5 and ML11 expression, their association in leukemia cells and functional annotation analysis of WDR5 target genes. (**A**) WDR5 and MLL1 expression in RS4;11, THP1, Nalm6 and U937 cells. Cells were harvested, and the cells were lysed with 2xSDS sample buffer and boiled for 10 min. The expression of WDR5, MLL1 and PCNA were detected by western blot with their specific antibodies. (**B**) The association of WDR5 with MLL was identified in RS4;11 cells by co-IP. (**C**) The WDR5 target genes were analyzed with Functional Annotation and Classification, David Bioinformatics Resources 6.7 in RS4;11 and THP-1 cells. The significant altered GO term and pathways were obtained. This figure showed the percentage of the enriched genes occupied all genes in each GO term or pathways.

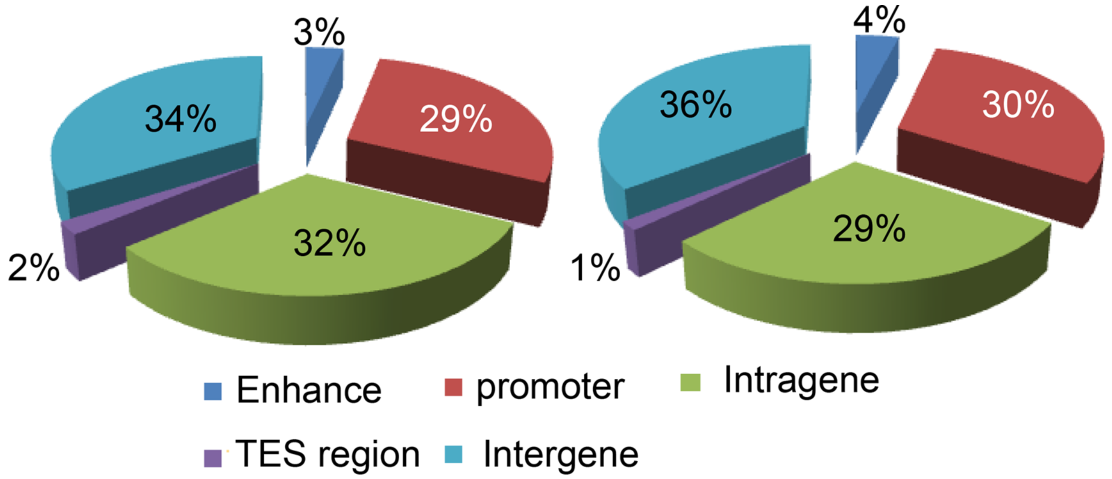

Supplementary Figure S2: Genome-wide distribution of H3K4me3 peaks. Percentages of peaks that bind sites in each location are given.

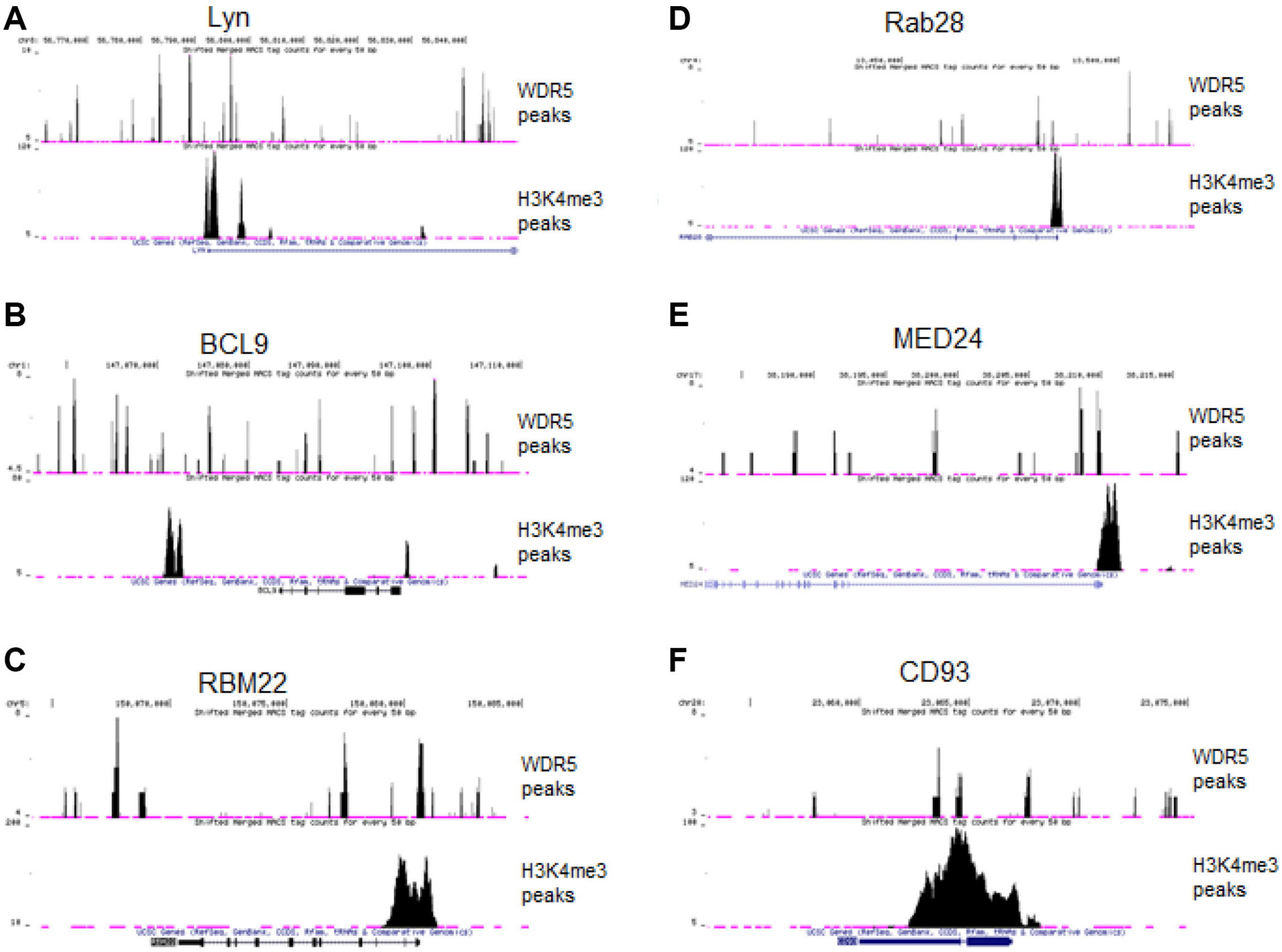

Supplementary Figure S3: Representative WDR5 and H3K4me3 binding peaks identified by ChIP-seq analysis in THP-1 cells. WDR5 and WDR5 signal maps are shown for representative target genes that are involved in cell proliferation and apoptosis regulation.

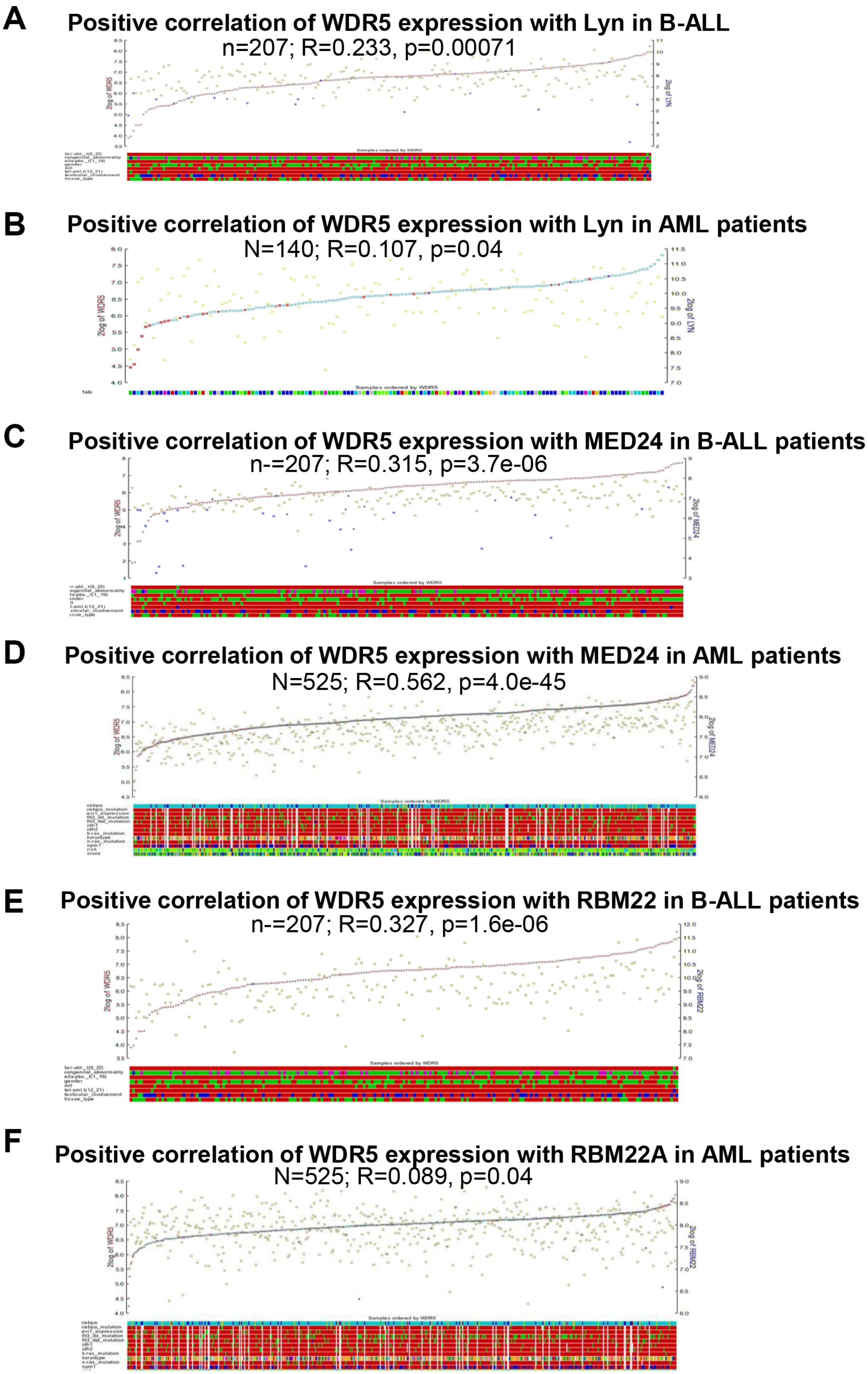

Supplementary Figure S4: Correlation of WDR5 expression with its gene targets in B-ALL and AML cohort. (**A–F**) Correlation of WDR5 expression with Lyn, MED24 or RBM22 in B-ALL (A, C,E) and AML (B,D,F) cohort. Data generated from human oncogenomics sever with GEO database (GSE11877) for B-ALL and (GPL97) for AML. Pearson correlation is used to calculate the statistics. The $P < 0.05$ was considered statistically significant.

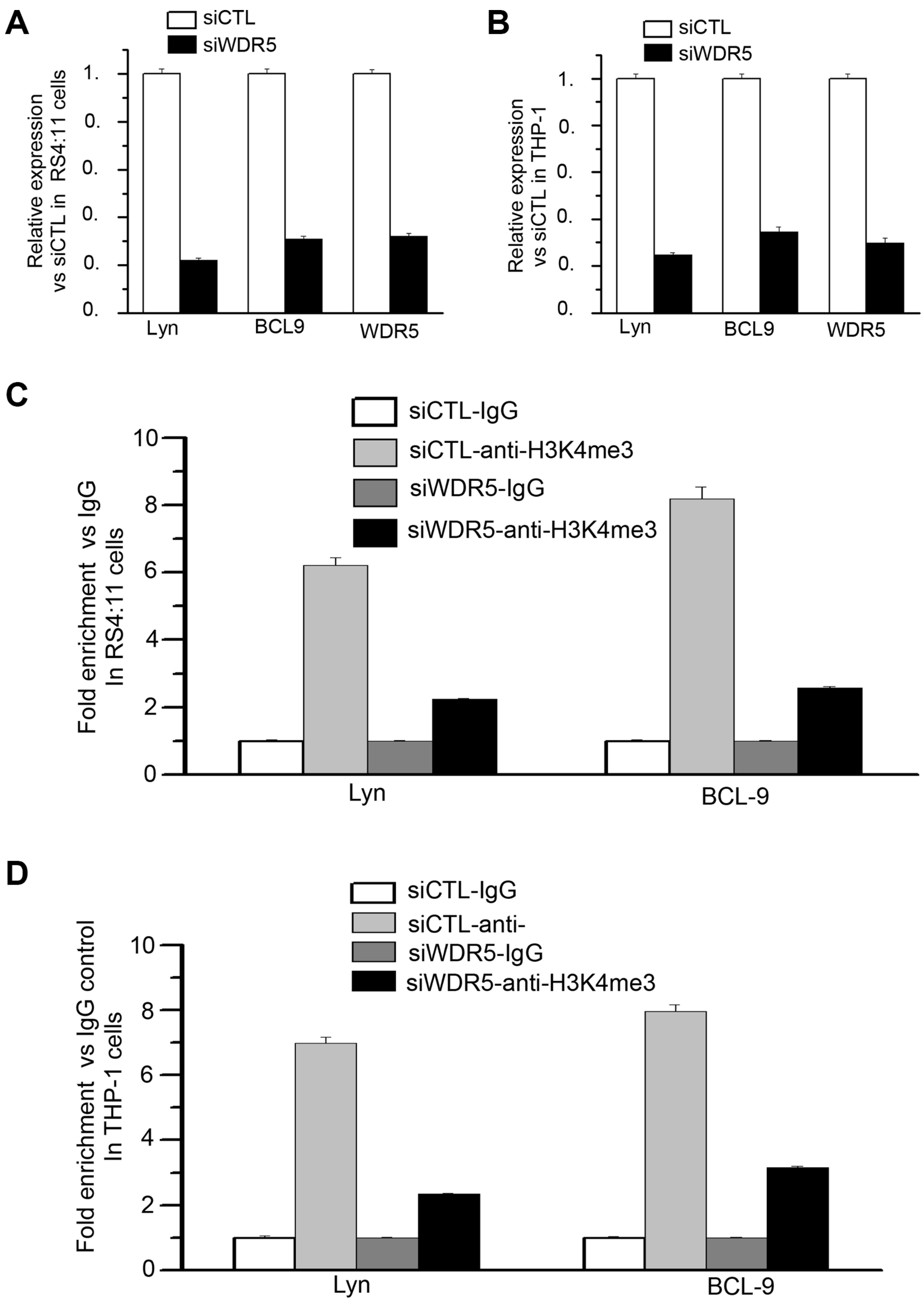

Supplementary Figure S5: WDR knockdown suppress the expression of its targets and H3K4me3 binding to the promoter of its targets. (**A–B**) Lyn and BCL9 expression in RS4:11 (A) and THP-1 (B) cells. Cells treated with siWDR5 shRNA and scramble shRNA for two days, total RNA was isolated for gene expression by qPCR. (**C–D**) H3K4me3 binding on Lyn and BCL9 in RS4:11 (C) and THP-1 (D) cells. Cells treated with siWDR5 shRNA and scramble shRNA for two days, qChIP assay was performed for the binding with anti-H3K4me3 antibody.

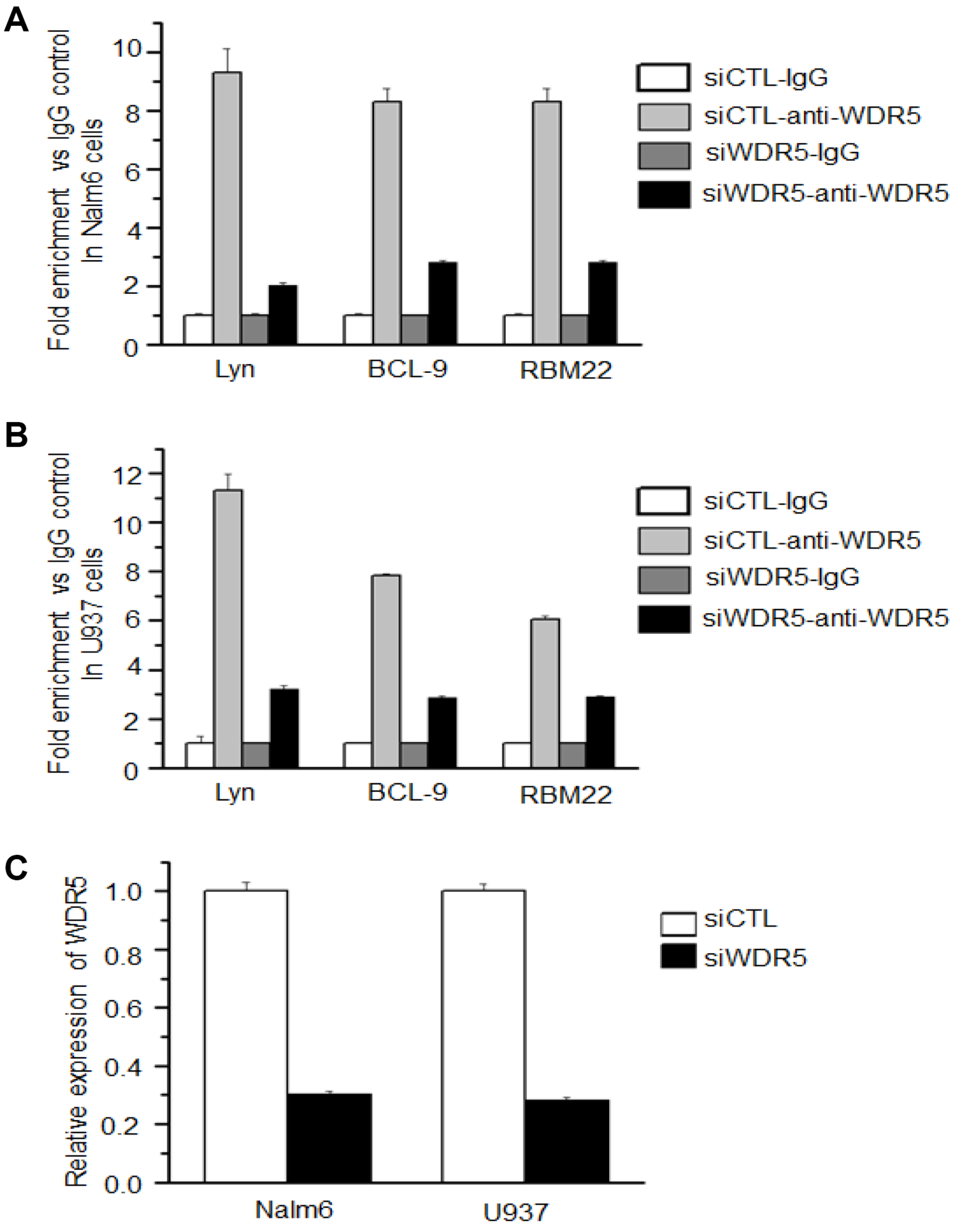

Supplementary Figure S6: WDR knockdown blocks its binding to the promoter of the gene targets. (**A–B**) WDR5 binding on Lyn, BCL9 and RBM22 in Nalm6 (A) and U937 (B) cells. Cells treated with siWDR shRNA and siCTL for two days, qChIP assay was performed for the binding with anti-WDR5 antibody. (**C**) It shows the efficiency of WDR5 knockdown by qPCR..

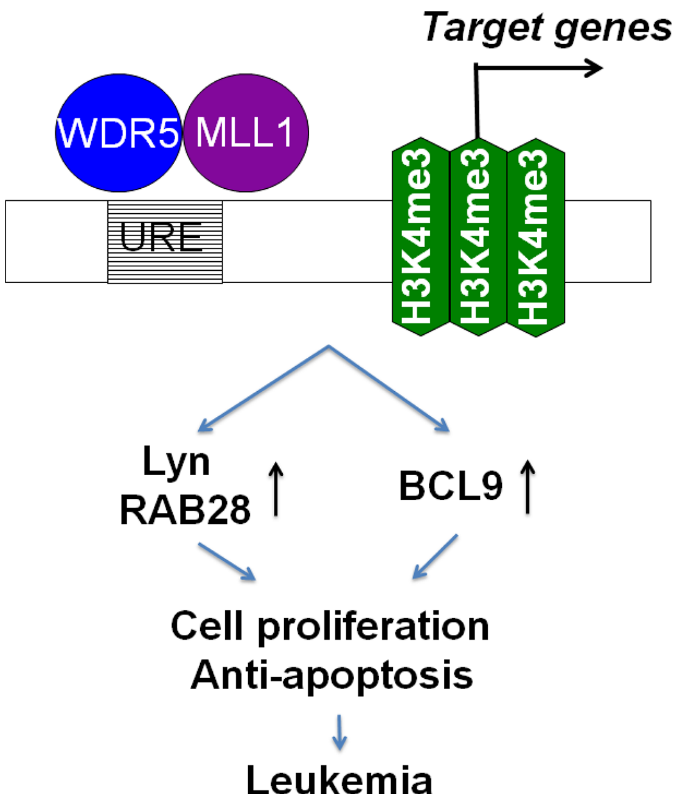

Supplementary Figure S7: Model for oncogenic effect of WDR5 high expression.

Supplementary Table S1: Correlation of *WDR5* and *MLL*1 expression with clinical features in adult ALL

	WDR5 expression			MLL1 expression		
**Characteristics**	**Low (*n* = 15)**	**High (*N* = 45)**	***P* value**	**Low (*n* = 27)**	**High (*n* = 30)**	***P* value**
**Age (years)**						
**Median (range)**	39 (14–52)	36 (14–77)	0.758	35 (14–77)	39.5 (16–75)	0.192
**Sex (%)**						
**male**	46.7	64.4	0.224	51.9	70.0	0.160
**WBC (×10^9/L)**						
**Median (range)**	47.3 (2.9–237.7)	74.0 (1.0–398.0)	0.567	46.7 (2.0–238.0)	69.7 (1.2–398.4)	0.755
**HGB (g/L)**						
**Median (range)**	113.0 (69.0–171.0)	92.0 (28.0–150.0)	0.092	113.5 (44.0–171.0)	90.0 (28.0–150.0)	0.120
**PLT (×10^9/L)**						
**Median (range)**	36.0 (22.0–219.0)	37.0 (5.0–212.0)	0.990	34.0 (5.0–219.0)	41.5 (9.0–211.0)	0.706
**LDH (μ/L)**						
**Median (range)**	1375.0 (511.0–2160.0)	880.0 (214.0–7142.0)	0.671	2160.0 (511.0–7142.0)	477.5 (214.0–3224.0)	0.051
**Blasts (%) median (range)**						
**Bone marrow**	72.4 (32.0–93.0)	87.6 (22.4–99.5)	0.022	74.4 (22.4–99.5)	87.8 (24.0–98.8)	0.058
**Peripheral blood**	77.5 (25.0–91.0)	57.0 (0.0–97.0)	0.210	70.0 (0.0–91.0)	68.0 (0.0–97.0)	0.927
**Immunopenotypic subtype**						
**B-ALL (%)**	40.0	75.6	0.011	59.3	70.0	0.396
**T-ALL (%)**	60.0	24.4	0.011	40.7	30.0	0.396
**Immune phenotype,%**						
**CD34+**	53.8	60.7	0.678	47.4	65.0	0.341
**HLA-DR+**	50.0	81.5	0.061	72.2	68.4	1.000
**CD10+**	75.0	75.0	1.000	77.8	70.0	0.719
**CD19+**	41.7	74.2	0.099	60.0	66.7	0.658
**CD20+**	0.0	60.0	0.001	18.8	57.9	0.036
**CD22+**	36.4	74.1	0.061	58.8	63.2	1.000
**CD13+**	45.5	50.0	1.000	43.8	53.3	0.724
**CD33+**	41.7	54.2	0.725	41.2	58.8	0.494
**CD2+**	55.6	19.0	0.082	46.2	18.8	0.226
**CD3+**	18.2	37.0	0.444	36.8	27.8	0.728
**CD5+**	90.0	44.4	0.041	71.4	53.8	0.440
**CD7+**	75.0	34.8	0.035	55.6	43.8	0.732
**CD38**	87.5	91.7	1.000	87.5	90.9	1.000
**Extramedullary infiltration (%)**						
**liver**	13.3	51.7	0.013	16.7	63.2	0.002
**spleen**	20.0	72.4	0.001	37.5	73.7	0.018
**Ph chromosome positive (%)**	0.0	34.4	0.026	8.7	31.8	0.117
***MLL*1 high expression (%)**	13.3	66.7	0.000			
***WDR5* high expression (%)**				51.9	93.3	0.000

Supplementary Table S2: Correlation of *WDR5* and *MLL*1 expression with clinical features in adult AML

	WDR5 expression			MLL1 expression		
Characteristics	Low (n = 61)	High (n = 27)	P value	Low (n = 48)	High (n = 43)	P value
**Age (years)**						
**Median (range)**	43 (14–75)	47 (15–73)	0.664	39.5 (14–75)	45 (15–74)	0.149
**Sex (%)**						
**male**	54.1	51.9	0.846	52.1	53.5	0.893
**WBC(×10^9/L)**						
**Median (range)**	26.6 (0.9–355.6)	40.0 (2.1–276.1)	0.507	17.9 (0.7–355.6)	40.0 (1.4–278.0)	0.289
**HGB (g/L)**						
**Median (range)**	90.5 (36.0–137.0)	87.5 (65.0–142.0)	0.835	90.0 (36.0–137.0)	88.5 (44.0–142.0)	0.834
**PLT (×10^9/L)**						
**Median (range)**	40.0 (7.0–207.0)	26.5 (4.0–203.0)	0.358	39.0 (10.0–207.0)	40.0 (4.0–203.0)	0.679
**LDH (μ/L)**						
**Median (range)**	423.0 (131.0–4434.0)	544.0 (120.0–2455.0)	0.634	430.0 (131.0–4434.0)	490.5 (120.0–2455.0)	0.865
**Blasts (%), median(range)**						
**Bone marrow**	77.9 (20.4–95.2)	90.8 (23.6–96.0)	0.008	75.2 (20.4–93.6)	85.4 (23.6–96.0)	0.011
**Peripheral blood**	65.5 (2.0–97.0)	81.5 (4.0–98.0)	0.049	65.0 (2.0–93.0)	77.5 (3.6–98.0)	0.156
**Morphologic subtype (%)**			0.430			0.526
**M0**	0.0	3.7		2.3	0.0	
**M1**	24.1	33.3		18.2	35.0	
**M2**	31.5	18.5		29.5	25.0	
**M3**	16.7	11.1		20.5	12.5	
**M4**	14.8	11.1		15.9	10.0	
**M5**	11.1	14.8		11.4	12.5	
**M6**	1.9	7.4		2.3	5.0	
**Risk status (%)**						
**Better-risk**[#]	54.7	22.2	0.016	61.0	31.0	0.023
**Intermediate-risk**[##]	43.4	70.4	< 0.001	36.6	64.3	< 0.001
**Poor-risk**[###]	1.9	7.4	< 0.001	2.4	4.8	< 0.001
***MLL*1 high expression (%)**	31.1	85.2	0.000			
***WDR5* high expression (%)**				8.7	54.8	0.000

[#]Favorable-risk: inv(16) or t(16;16); t(8;21); t(15;17); normal cytogenetics with NPM1 mutation or isolated CEBPA mutation in the absence of FLT3. [##]Intermediate-risk: Normal cytogenetics; +8; t(9;11); Other non-defined; t(8;21), inv (16), t(16;16) with c-KITmutation. [###]Poor-risk: Complex (3 abnormal clones); -5, 5q-, -7, 7q-; 11q23 - non t(9;11); inv(3), t(3;3); t(6;9); t(9;22); Normal cytogenetics with FLT3-ITD mutation.

Supplementary Table S3: Correlation of high expression of both *MLL*1 and *WDR5* with clinical features in adult ALL and AML

	ALL		
	MLL1* and *WDR5		
**Characteristics**	***WDR5*^{low} *MLL*^{low} (*n* = 13)**	***WDR5*^{high}*MLL*^{high} (*n* = 28)**	***P* value**
**Age (years)**			
**Median (range)**	35.0 (14–50)	37.5 (16–75)	0.324
**Sex (%)**			
**male**	53.8	75.0	0.320
**WBC (×10^9/L)**			
**Median (range)**	46.7 (2.9–237.7)	71.4 (1.2–398.4)	0.730
**HGB (g/L)**			
**Median (range)**	123.0 (69.0–171.0)	90.0 (28.0–150.0)	0.029
**PLT (×10^9/L)**			
**Median (range)**	32.0 (22.0–219.0)	46.5 (9.0–211.0)	0.848
**LDH (μ/L)**			
**Median (range)**	1375.0 (511.0–2160.0)	477.5 (214.0–3224.0)	
**Blasts (%) median (range)**			
**Bone marrow**	62.0 (32.0–93.0)	87.8 (24.0–98.8)	0.011
**Peripheral blood**	77.5 (25.0–91.0)	59.0 (0.0–97.0)	0.481
**Immunopenotypic subtype**			
**B-ALL (%)**	46.2	75.0	0.145
**T-ALL (%)**	53.8	25.0	0.145
**Immune phenotype (%)**			
**CD34+**	54.5	66.7	0.696
**HLA-DR+**	60.0	76.5	0.415
**CD10+**	80.0	72.2	1.000
**CD19+**	50.0	73.7	0.244
**CD20+**	0.0	64.7	0.002
**CD22+**	44.4	70.6	0.234
**CD13+**	33.3	46.2	0.674
**CD33+**	40.0	60.0	0.428
**CD2+**	57.1	14.3	0.120
**CD3+**	22.2	31.3	1.000
**CD5+**	87.5	45.5	0.147
**CD7+**	70.0	35.7	0.214
**CD38**	83.3	88.9	1.000
**Extramedullary infiltration (%)**			
**liver**	0.0	58.8	0.001
**spleen**	15.4	76.5	0.003
**Ph chromosome positive (%)**	0.0	35.0	0.027

	AML		
	MLL1* and *WDR5		
**Characteristics**	***WDR5*^{low}*MLL*^{low} (*n* = 42)**	***WDR5*^{high}*MLL*^{high} (*n* = 23)**	***P* value**
**Age (years)**			
**Median (range)**	39 (14–75)	47 (15–73)	0.387
**Sex (%)**			
**male**	54.8	56.5	0.891

**WBC ($\times 10^9$/L)**			
**Median(range)**	21.8 (0.9–355.6)	40.0 (2.1–276.1)	0.507
**HGB (g/L)**			
**Median(range)**	88.0 (36.0–137.0)	84.0 (65.0–142.0)	0.876
**PLT ($\times 10^9$/L)**			
**Median (range)**	39.0 (10.0–207.0)	20.0 (4.0–203.0)	0.326
**LDH (μ/L)**			
**Median(range)**	423.0 (131.0–4434.0)	552.5 (120.0–2455.0)	0.892
**Blasts (%) median(range)**			
**Bone marrow**	68.5 (20.4–93.6)	90.0 (23.6–96.0)	0.004
**Peripheral blood**	65.0 (2.0–93.0)	81.0 (4.0–98.0)	0.066
**Morphologic subtype (%)**			0.453
**M1**	18.4	34.8	
**M2**	31.6	17.4	
**M3**	15.8	8.7	
**M4**	18.4	13.0	
**M5**	13.2	17.4	
**M6**	2.6	8.7	
**Risk status (%)**			
**Better-risk[#]**	62.9	21.7	0.006
**Intermediate-risk[##]**	37.1	73.9	< 0.001
**Poor-risk[###]**	0.0	4.3	< 0.001

[#]Favorable-risk: inv(16) or t(16;16); t(8;21); t(15;17); normal cytogenetics with NPM1 mutation or isolated CEBPA mutation in the absence of FLT3. [##]Intermediate-risk: Normal cytogenetics; +8; t(9;11); Other non-defined; t(8;21), inv (16), t(16;16) with c-KITmutation. [###]Poor-risk: Complex (3 abnormal clones); -5, 5q-, -7, 7q-; 11q23 - non t(9;11); inv(3), t(3;3); t(6;9); t(9;22); Normal cytogenetics with FLT3-ITD mutation.

Supplementary Table S4: Primers for qRT-PCR of *WDR5* target genes and qChIP of WDR5 and H3K4me3 binding

A Primers for qRT-PCR of *WDR5* target genes in oncogenesis, apoptosis and others

Lyn-F	5′- GAGCGATGAAGGTGGCAAAG-3′	Lyn-R	5′- TGCAATCTGAGCAGAAAAGTCAA -3′
BCL9-F	5′-GAAATGGAAGGGCCGAATG-3′	BCL9-R	5′-GGCCAACTGACTCCAGAAAGAC -3′
CD93-F	5′-CCAGAATGCGGCAGACAGTT-3′	CD93-R	5′-TCCATGGCCCTGCTCTCA-3′
MED24-F	5′-CCTTCGAGTCCATCCAGAAAAT-3′	MED24-R	5′-CACCGCCAGACTGCATACC-3′
RBM22-F	5′-TGGGAAGGAATGCAAAATCTG-3′	RBM22-R	5′-AGGGCACCAGCGAAACAC-3′
PIGK-F	5′-TGGGCTGTTCTGGTGTGTACA-3′	PIGK-R	5′-AAGGGTATTTGCAACATGTCGAT-3′

B Primers for qChIP analysis of *WDR5* and H3K4me3 binding at *WDR5* genes targets on oncogenesis, apoptosis and others

Lyn-F	5′- CACTTCACTTTCACAAGTG-3′	Lyn-R	5′-gattagagtggtgaatgtaac -3′
BCL9-F	5′- TCTCCAGCTAATGGGACTTTGC-3′	BCL9-R	5′- CAGCGCCAGGAAAAGTGAAA-3′
CD93-F	5′- ATGAACAACAGCACCGTGATG-3′	CD93-R	5′- GGGCTTCCTGCAATGAATAGTT-3′
MED24-F	5′- GAATGAAAGGGTGTCGGAATATG -3′	MED24-R	5′- TCGCCCACGACGTCACT-3′
RBM22-F	5′- ATAAGACACTGGAACCGCAAGAC-3′	RBM22-R	5′- CCACTGAGGCGCTTATTATGC-3′
PIGK-F	5′- CTCCACAAAACCGCAGAGTTC-3′	PIGK-R	5′- ACCATCCGCGCCTCTTC-3′

C Primers for qChIP analysis of H3K4me3 binding in *WDR5* targets

Lyn-F1	5′-CTGTATGAGATGCAAAGGCAAC-3′	Lyn-R1	5′-GCAACTATAATTATAACG-3′
Lyn-F2	5′-GGCTGGTCTCAAACTCCTAACCT-3′	Lyn-R2	5′-TCCTGTAATCCCAGCACTTTGG-3′
Lyn-F3	5′-CACTTCACTTTCACAAGTG-3′	Lyn-R3	5′-GATTAGAGTGGTGAATGTAAC-3′
Lyn-F4	5′-TGGTTGGCACGTAATGAGAAAA-3′	Lyn-R4	5′-TTGACCCCAGAGCTCATGATC-3′
Lyn-F5	5′-CTCACGCCTCTAATCCCAGCAC-3′	Lyn-R5	5′-CAGGCTGGTCTCGAACTCCTGAC-3′
Lyn-F6	5′-GTTGTAGTATTATCATACAGAG-3′	Lyn-R6	5′-GCTTCATCTGTTTGAAGCTATG-3′
BCL9-F1	5′-GTACCTGGACTACTCCAGTG-3′	BCL9-R1	5′-CAACTGAGGCCTGTGTGATTC-3′
BCL9-F2	5′-CTATGAAGGAATGAGTGATGTGTG-3′	BCL9-R2	5′-GTTTAGACTCTATTCCTCAG -3′
BCL9-F3	5′-CTTGTTTCTTGCCATATTCATTATC-3′	BCL9-R3	5′-GAGGGGAGGGTGAGTGTGCTG-3′
BCL9-F4	5′-CTGGCGCTGAGATGGCCTGAG-3′	BCL9-R4	5′-CAGAAGAGAGGTGAGTCCTTG-3′
BCL9-F5	5′-GAGAATGTCTTGTTTGTGGCTTG-3′	BCL9-R5	5′-GTACGGCCCAGGCATTCATGCCAAGTAG-3′
BCL9-F6	5′-GACTGGGGATGGCCCCTCTTTC-3′	BCL9-R6	5′-CTCTCCATCCACCCCTTCTC-3′
